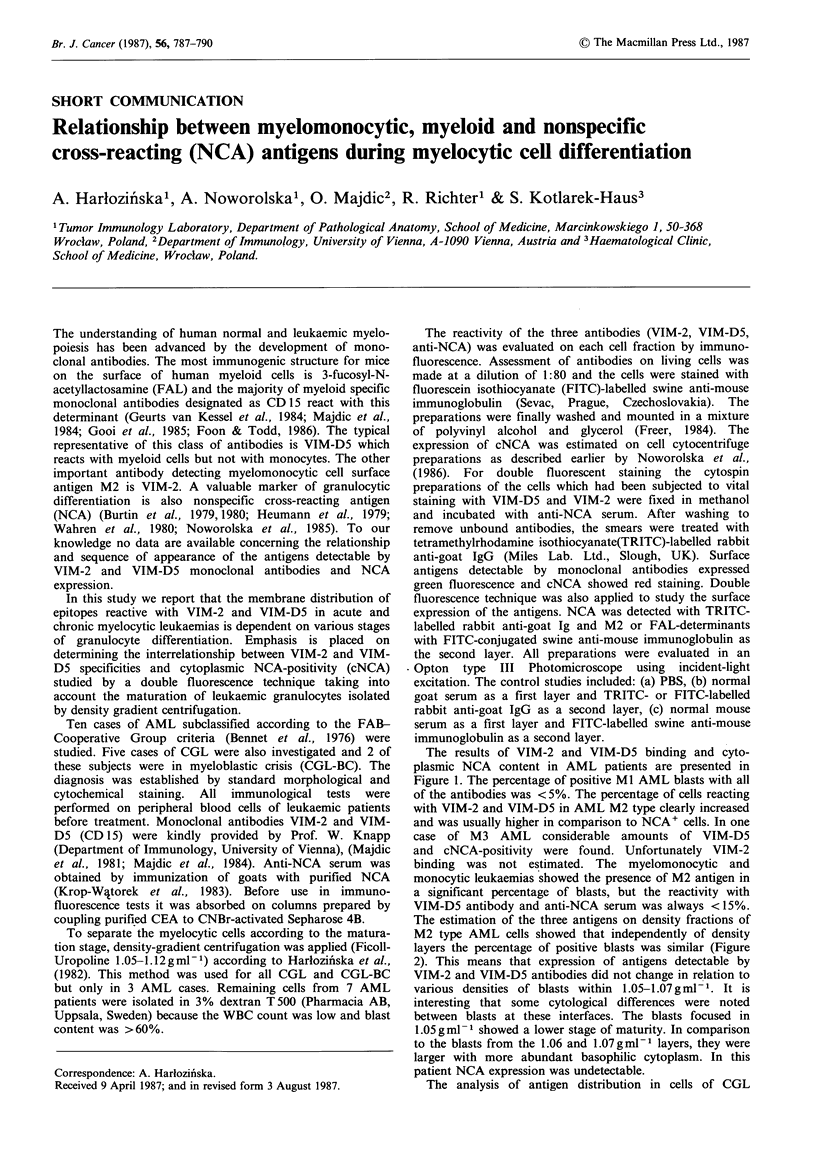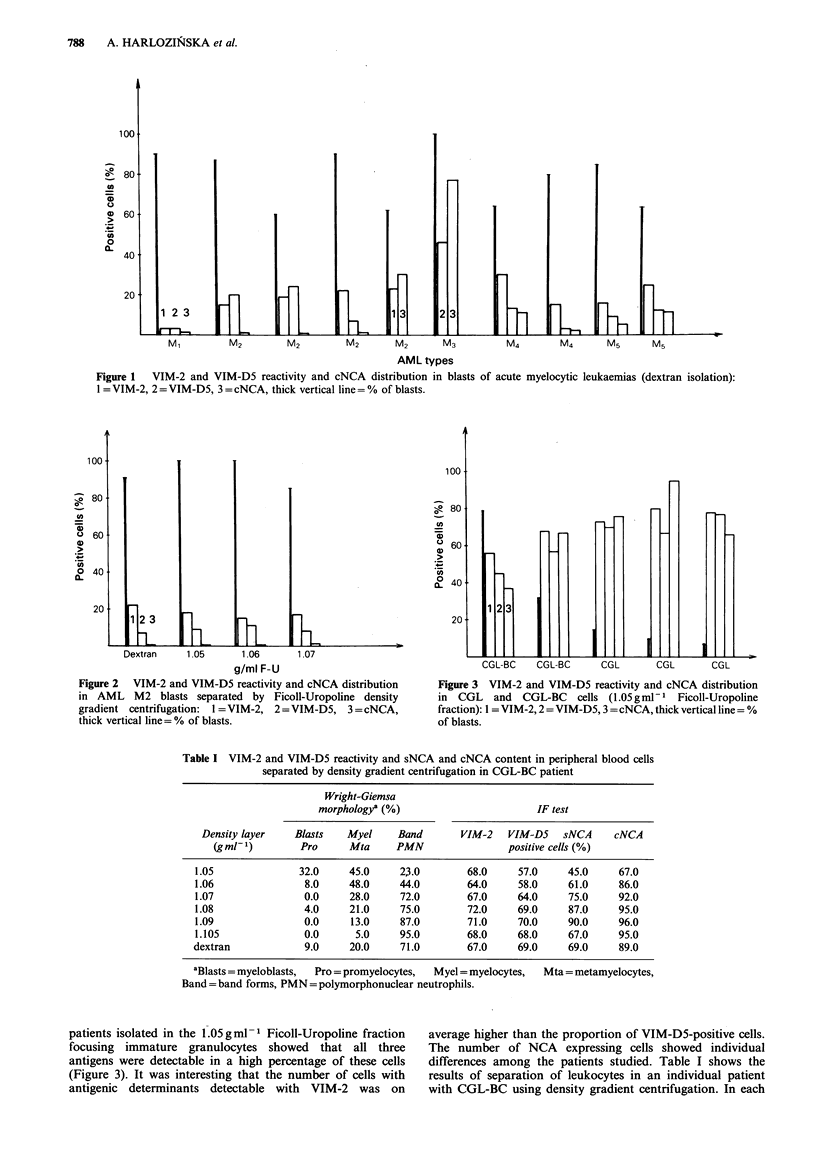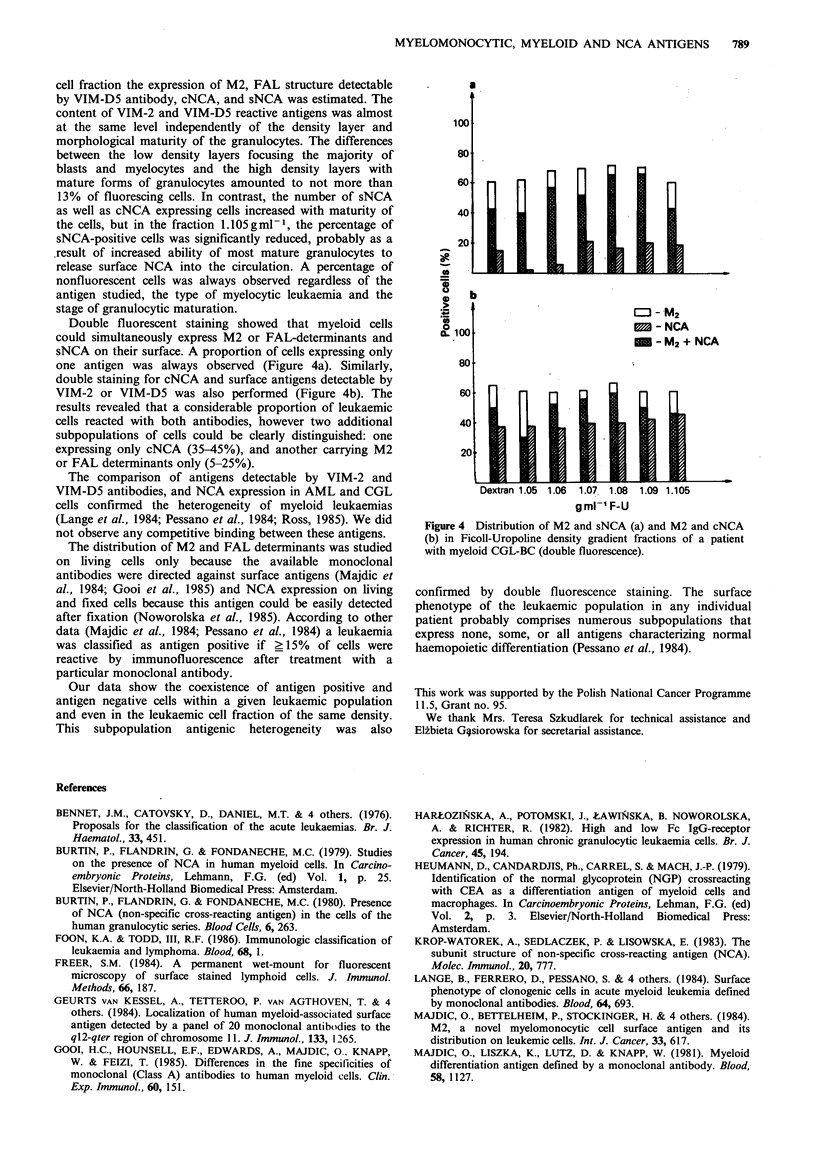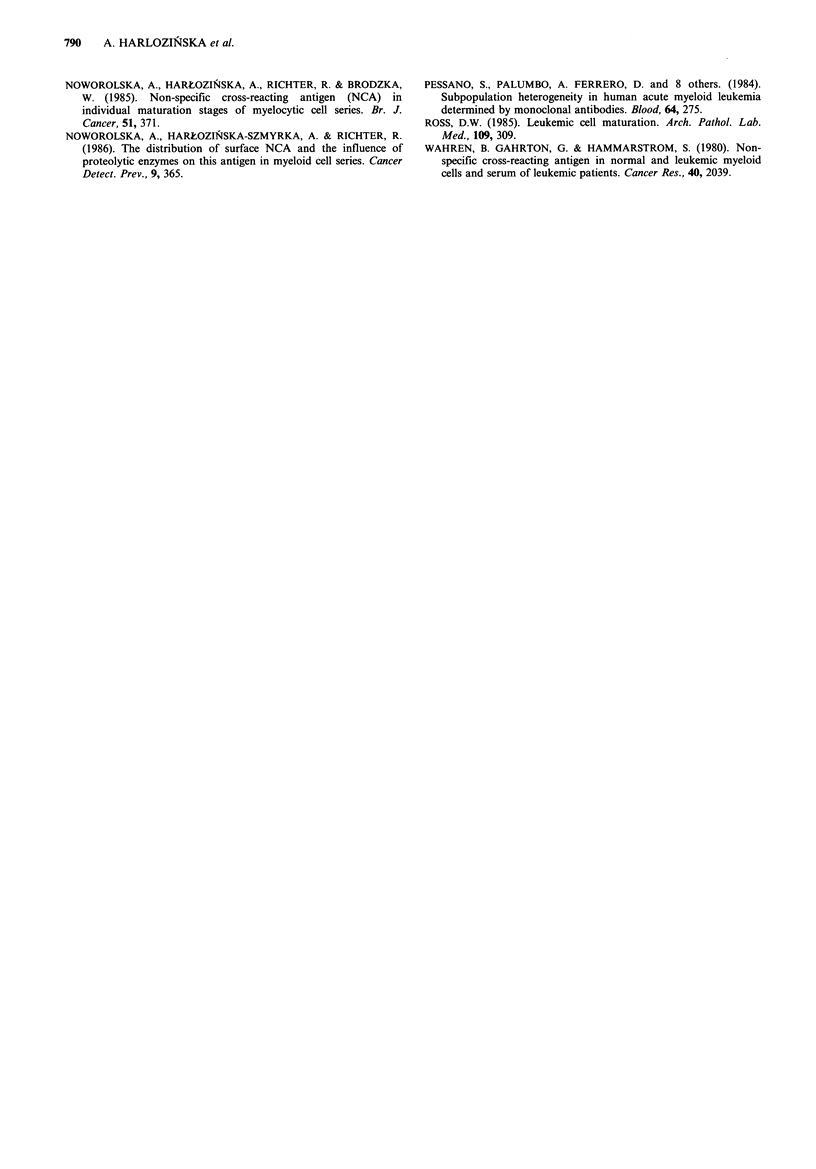# Relationship between myelomonocytic, myeloid and nonspecific cross-reacting (NCA) antigens during myelocytic cell differentiation.

**DOI:** 10.1038/bjc.1987.289

**Published:** 1987-12

**Authors:** A. Harłozińska, A. Noworolska, O. Majdic, R. Richter, S. Kotlarek-Haus

**Affiliations:** Department of Pathological Anatomy, School of Medicine, Wrocław, Poland.


Br. J. Cancer (1987), 56, 787–790 © The Macmillan Press Ltd., 1987

SHORT COMMUNICATION

### Relationship between myelomonocytic, myeloid and nonspecific cross-reacting (NCA) antigens during myelocytic cell differentiation

A. Harłozińska[1], A. Noworolska[1], O. Majdic[2], R. Richter[1] & S. Kotlarek-Haus[3]

[1]*Tumor Immunology Laboratory, Department of Pathological Anatomy, School of Medicine, Marcinkowskiego 1, 50-368 Wrocław, Poland,* [2]*Department of Immunology, University of Vienna, A-1090 Vienna, Austria and* [3]*Haematological Clinic, School of Medicine, Wrocław, Poland.*

The understanding of human normal and leukaemic myelopoiesis has been advanced by the development of monoclonal antibodies. The most immunogenic structure for mice on the surface of human myeloid cells is 3-fucosyl-N-acetyllactosamine (FAL) and the majority of myeloid specific monoclonal antibodies designated as CD 15 react with this determinant (Geurts van Kessel *et al.*, 1984; Majdic *et al.*, 1984; Gooi *et al.*, 1985; Foon & Todd, 1986). The typical representative of this class of antibodies is VIM-D5 which reacts with myeloid cells but not with monocytes. The other important antibody detecting myelomonocytic cell surface antigen M2 is VIM-2. A valuable marker of granulocytic differentiation is also nonspecific cross-reacting antigen (NCA) (Burtin *et al.*, 1979, 1980; Heumann *et al.*, 1979; Wahren *et al.*, 1980; Noworolska *et al.*, 1985). To our knowledge no data are available concerning the relationship and sequence of appearance of the antigens detectable by VIM-2 and VIM-D5 monoclonal antibodies and NCA expression.

In this study we report that the membrane distribution of epitopes reactive with VIM-2 and VIM-D5 in acute and chronic myelocytic leukaemias is dependent on various stages of granulocyte differentiation. Emphasis is placed on determining the interrelationship between VIM-2 and VIM-D5 specificities and cytoplasmic NCA-positivity (cNCA) studied by a double fluorescence technique taking into account the maturation of leukaemic granulocytes isolated by density gradient centrifugation.

Ten cases of AML subclassified according to the FAB-Cooperative Group criteria (Bennet *et al.*, 1976) were studied. Five cases of CGL were also investigated and 2 of these subjects were in myeloblastic crisis (CGL-BC). The diagnosis was established by standard morphological and cytochemical staining. All immunological tests were performed on peripheral blood cells of leukaemic patients before treatment. Monoclonal antibodies VIM-2 and VIM-D5 (CD 15) were kindly provided by Prof. W. Knapp (Department of Immunology, University of Vienna), (Majdic *et al.*, 1981; Majdic *et al.*, 1984). Anti-NCA serum was obtained by immunization of goats with purified NCA (Krop-Wątorek *et al.*, 1983). Before use in immunofluorescence tests it was absorbed on columns prepared by coupling purified CEA to CNBr-activated Sepharose 4B.

To separate the myelocytic cells according to the maturation stage, density-gradient centrifugation was applied (Ficoll-Uropoline 1.05–1.12 g ml$^{-1}$) according to Harłozińska *et al.*, (1982). This method was used for all CGL and CGL-BC but only in 3 AML cases. Remaining cells from 7 AML patients were isolated in 3% dextran T 500 (Pharmacia AB, Uppsala, Sweden) because the WBC count was low and blast content was >60%.

The reactivity of the three antibodies (VIM-2, VIM-D5, anti-NCA) was evaluated on each cell fraction by immunofluorescence. Assessment of antibodies on living cells was made at a dilution of 1:80 and the cells were stained with fluorescein isothiocyanate (FITC)-labelled swine anti-mouse immunoglobulin (Sevac, Prague, Czechoslovakia). The preparations were finally washed and mounted in a mixture of polyvinyl alcohol and glycerol (Freer, 1984). The expression of cNCA was estimated on cell cytocentrifuge preparations as described earlier by Noworolska *et al.*, (1986). For double fluorescent staining the cytospin preparations of the cells which had been subjected to vital staining with VIM-D5 and VIM-2 were fixed in methanol and incubated with anti-NCA serum. After washing to remove unbound antibodies, the smears were treated with tetramethylrhodamine isothiocyanate(TRITC)-labelled rabbit anti-goat IgG (Miles Lab. Ltd., Slough, UK). Surface antigens detectable by monoclonal antibodies expressed green fluorescence and cNCA showed red staining. Double fluorescence technique was also applied to study the surface expression of the antigens. NCA was detected with TRITC-labelled rabbit anti-goat Ig and M2 or FAL-determinants with FITC-conjugated swine anti-mouse immunoglobulin as the second layer. All preparations were evaluated in an Opton type III Photomicroscope using incident-light excitation. The control studies included: (a) PBS, (b) normal goat serum as a first layer and TRITC- or FITC-labelled rabbit anti-goat IgG as a second layer, (c) normal mouse serum as a first layer and FITC-labelled swine anti-mouse immunoglobulin as a second layer.

The results of VIM-2 and VIM-D5 binding and cytoplasmic NCA content in AML patients are presented in Figure 1. The percentage of positive M1 AML blasts with all of the antibodies was <5%. The percentage of cells reacting with VIM-2 and VIM-D5 in AML M2 type clearly increased and was usually higher in comparison to NCA$^+$ cells. In one case of M3 AML considerable amounts of VIM-D5 and cNCA-positivity were found. Unfortunately VIM-2 binding was not estimated. The myelomonocytic and monocytic leukaemias showed the presence of M2 antigen in a significant percentage of blasts, but the reactivity with VIM-D5 antibody and anti-NCA serum was always <15%. The estimation of the three antigens on density fractions of M2 type AML cells showed that independently of density layers the percentage of positive blasts was similar (Figure 2). This means that expression of antigens detectable by VIM-2 and VIM-D5 antibodies did not change in relation to various densities of blasts within 1.05–1.07 g ml$^{-1}$. It is interesting that some cytological differences were noted between blasts at these interfaces. The blasts focused in 1.05 g ml$^{-1}$ showed a lower stage of maturity. In comparison to the blasts from the 1.06 and 1.07 g ml$^{-1}$ layers, they were larger with more abundant basophilic cytoplasm. In this patient NCA expression was undetectable.

The analysis of antigen distribution in cells of CGL

Correspondence: A. Harłozińska.
Received 9 April 1987; and in revised form 3 August 1987.

**Figure 1** VIM-2 and VIM-D5 reactivity and cNCA distribution in blasts of acute myelocytic leukaemias (dextran isolation): 1 = VIM-2, 2 = VIM-D5, 3 = cNCA, thick vertical line = % of blasts.

**Figure 2** VIM-2 and VIM-D5 reactivity and cNCA distribution in AML M2 blasts separated by Ficoll-Uropoline density gradient centrifugation: 1 = VIM-2, 2 = VIM-D5, 3 = cNCA, thick vertical line = % of blasts.

**Figure 3** VIM-2 and VIM-D5 reactivity and cNCA distribution in CGL and CGL-BC cells (1.05 g ml$^{-1}$ Ficoll-Uropoline fraction): 1 = VIM-2, 2 = VIM-D5, 3 = cNCA, thick vertical line = % of blasts.

**Table I** VIM-2 and VIM-D5 reactivity and sNCA and cNCA content in peripheral blood cells separated by density gradient centrifugation in CGL-BC patient

| | *Wright-Giemsa morphology*[a] (%) | | | *IF test* | | | |
|---|---|---|---|---|---|---|---|
| *Density layer* ($g\,ml^{-1}$) | *Blasts Pro* | *Myel Mta* | *Band PMN* | *VIM-2* | *VIM-D5* (positive cells (%)) | *sNCA* (positive cells (%)) | *cNCA* |
| 1.05 | 32.0 | 45.0 | 23.0 | 68.0 | 57.0 | 45.0 | 67.0 |
| 1.06 | 8.0 | 48.0 | 44.0 | 64.0 | 58.0 | 61.0 | 86.0 |
| 1.07 | 0.0 | 28.0 | 72.0 | 67.0 | 64.0 | 75.0 | 92.0 |
| 1.08 | 4.0 | 21.0 | 75.0 | 72.0 | 69.0 | 87.0 | 95.0 |
| 1.09 | 0.0 | 13.0 | 87.0 | 71.0 | 70.0 | 90.0 | 96.0 |
| 1.105 | 0.0 | 5.0 | 95.0 | 68.0 | 68.0 | 67.0 | 95.0 |
| dextran | 9.0 | 20.0 | 71.0 | 67.0 | 69.0 | 69.0 | 89.0 |

[a]Blasts = myeloblasts, Pro = promyelocytes, Myel = myelocytes, Mta = metamyelocytes, Band = band forms, PMN = polymorphonuclear neutrophils.

patients isolated in the 1.05 g ml$^{-1}$ Ficoll-Uropoline fraction focusing immature granulocytes showed that all three antigens were detectable in a high percentage of these cells (Figure 3). It was interesting that the number of cells with antigenic determinants detectable with VIM-2 was on average higher than the proportion of VIM-D5-positive cells. The number of NCA expressing cells showed individual differences among the patients studied. Table I shows the results of separation of leukocytes in an individual patient with CGL-BC using density gradient centrifugation. In each cell fraction the expression of M2, FAL structure detectable by VIM-D5 antibody, cNCA, and sNCA was estimated. The content of VIM-2 and VIM-D5 reactive antigens was almost at the same level independently of the density layer and morphological maturity of the granulocytes. The differences between the low density layers focusing the majority of blasts and myelocytes and the high density layers with mature forms of granulocytes amounted to not more than 13% of fluorescing cells. In contrast, the number of sNCA as well as cNCA expressing cells increased with maturity of the cells, but in the fraction 1.105 g ml$^{-1}$, the percentage of sNCA-positive cells was significantly reduced, probably as a result of increased ability of most mature granulocytes to release surface NCA into the circulation. A percentage of nonfluorescent cells was always observed regardless of the antigen studied, the type of myelocytic leukaemia and the stage of granulocytic maturation.

Double fluorescent staining showed that myeloid cells could simultaneously express M2 or FAL-determinants and sNCA on their surface. A proportion of cells expressing only one antigen was always observed (Figure 4a). Similarly, double staining for cNCA and surface antigens detectable by VIM-2 or VIM-D5 was also performed (Figure 4b). The results revealed that a considerable proportion of leukaemic cells reacted with both antibodies, however two additional subpopulations of cells could be clearly distinguished: one expressing only cNCA (35–45%), and another carrying M2 or FAL determinants only (5–25%).

The comparison of antigens detectable by VIM-2 and VIM-D5 antibodies, and NCA expression in AML and CGL cells confirmed the heterogeneity of myeloid leukaemias (Lange *et al.*, 1984; Pessano *et al.*, 1984; Ross, 1985). We did not observe any competitive binding between these antigens.

The distribution of M2 and FAL determinants was studied on living cells only because the available monoclonal antibodies were directed against surface antigens (Majdic *et al.*, 1984; Gooi *et al.*, 1985) and NCA expression on living and fixed cells because this antigen could be easily detected after fixation (Noworolska *et al.*, 1985). According to other data (Majdic *et al.*, 1984; Pessano *et al.*, 1984) a leukaemia was classified as antigen positive if $\geq$15% of cells were reactive by immunofluorescence after treatment with a particular monoclonal antibody.

Our data show the coexistence of antigen positive and antigen negative cells within a given leukaemic population and even in the leukaemic cell fraction of the same density. This subpopulation antigenic heterogeneity was also confirmed by double fluorescence staining. The surface phenotype of the leukaemic population in any individual patient probably comprises numerous subpopulations that express none, some, or all antigens characterizing normal haemopoietic differentiation (Pessano *et al.*, 1984).

**Figure 4** Distribution of M2 and sNCA (a) and M2 and cNCA (b) in Ficoll-Uropoline density gradient fractions of a patient with myeloid CGL-BC (double fluorescence).

This work was supported by the Polish National Cancer Programme 11.5, Grant no. 95.

We thank Mrs. Teresa Szkudlarek for technical assistance and Elżbieta Gąsiorowska for secretarial assistance.

### References

BENNET, J.M., CATOVSKY, D., DANIEL, M.T. & 4 others. (1976). Proposals for the classification of the acute leukaemias. *Br. J. Haematol.*, **33**, 451.

BURTIN, P., FLANDRIN, G. & FONDANECHE, M.C. (1979). Studies on the presence of NCA in human myeloid cells. In *Carcinoembryonic Proteins*, Lehmann, F.G. (ed) Vol. **1**, p. 25. Elsevier/North-Holland Biomedical Press: Amsterdam.

BURTIN, P., FLANDRIN, G. & FONDANECHE, M.C. (1980). Presence of NCA (non-specific cross-reacting antigen) in the cells of the human granulocytic series. *Blood Cells*, **6**, 263.

FOON, K.A. & TODD, III, R.F. (1986). Immunologic classification of leukaemia and lymphoma. *Blood*, **68**, 1.

FREER, S.M. (1984). A permanent wet-mount for fluorescent microscopy of surface stained lymphoid cells. *J. Immunol. Methods*, **66**, 187.

GEURTS VAN KESSEL, A., TETTEROO, P. VAN AGTHOVEN, T. & 4 others. (1984). Localization of human myeloid-associated surface antigen detected by a panel of 20 monoclonal antibodies to the *q12-qter* region of chromosome 11. *J. Immunol.*, **133**, 1265.

GOOI, H.C., HOUNSELL, E.F., EDWARDS, A., MAJDIC, O., KNAPP, W. & FEIZI, T. (1985). Differences in the fine specificities of monoclonal (Class A) antibodies to human myeloid cells. *Clin. Exp. Immunol.*, **60**, 151.

HARŁOZIŃSKA, A., POTOMSKI, J., ŁAWIŃSKA, B. NOWOROLSKA, A. & RICHTER, R. (1982). High and low Fc IgG-receptor expression in human chronic granulocytic leukaemia cells. *Br. J. Cancer*, **45**, 194.

HEUMANN, D., CANDARDJIS, Ph., CARREL, S. & MACH, J.-P. (1979). Identification of the normal glycoprotein (NGP) crossreacting with CEA as a differentiation antigen of myeloid cells and macrophages. In *Carcinoembryonic Proteins*, Lehman, F.G. (ed) Vol. **2**, p. 3. Elsevier/North-Holland Biomedical Press: Amsterdam.

KROP-WATOREK, A., SEDLACZEK, P. & LISOWSKA, E. (1983). The subunit structure of non-specific cross-reacting antigen (NCA). *Molec. Immunol.*, **20**, 777.

LANGE, B., FERRERO, D., PESSANO, S. & 4 others. (1984). Surface phenotype of clonogenic cells in acute myeloid leukemia defined by monoclonal antibodies. *Blood*, **64**, 693.

MAJDIC, O., BETTELHEIM, P., STOCKINGER, H. & 4 others. (1984). M2, a novel myelomonocytic cell surface antigen and its distribution on leukemic cells. *Int. J. Cancer*, **33**, 617.

MAJDIC, O., LISZKA, K., LUTZ, D. & KNAPP, W. (1981). Myeloid differentiation antigen defined by a monoclonal antibody. *Blood*, **58**, 1127.

NOWOROLSKA, A., HARŁOZIŃSKA, A., RICHTER, R. & BRODZKA, W. (1985). Non-specific cross-reacting antigen (NCA) in individual maturation stages of myelocytic cell series. *Br. J. Cancer*, **51**, 371.

NOWOROLSKA, A., HARŁOZIŃSKA-SZMYRKA, A. & RICHTER, R. (1986). The distribution of surface NCA and the influence of proteolytic enzymes on this antigen in myeloid cell series. *Cancer Detect. Prev.*, **9**, 365.

PESSANO, S., PALUMBO, A. FERRERO, D. and 8 others. (1984). Subpopulation heterogeneity in human acute myeloid leukemia determined by monoclonal antibodies. *Blood*, **64**, 275.

ROSS, D.W. (1985). Leukemic cell maturation. *Arch. Pathol. Lab. Med.*, **109**, 309.

WAHREN, B. GAHRTON, G. & HAMMARSTROM, S. (1980). Non-specific cross-reacting antigen in normal and leukemic myeloid cells and serum of leukemic patients. *Cancer Res.*, **40**, 2039.